# A systematic review exploring the bidirectional relationship between puberty and autoimmune rheumatic diseases

**DOI:** 10.1186/s12969-021-00528-y

**Published:** 2021-03-29

**Authors:** Nina M. de Gruijter, Meena Naja, Hannah Peckham, Anna Radziszewska, Matthew Kinsella, James Glenister, Elizabeth C. Rosser, Gary E. Butler, Elizabeth C. Jury, Coziana Ciurtin

**Affiliations:** 1 Centre for Adolescent Rheumatology Versus Arthritis, Department of Medicine, University College London, Rayne Building, London, W1CE 6JF UK; 2 Centre for Rheumatology Research, Division of Medicine, University College London, London, UK; 3 University College London Medical School, London, UK; 4 Department of Paediatric & Adolescent Medicine and Endocrinology, University College London Hospital NHS Foundation Trust; University College London Great Ormond Street Institute of Child Health, London, UK

**Keywords:** Autoimmune rheumatic diseases, Puberty, Adolescence, Sex hormones, Systemic lupus erythematosus, Juvenile idiopathic arthritis

## Abstract

**Background:**

Autoimmune rheumatic diseases (ARDs) are associated with a significant sex-bias, which becomes more evident post-puberty. This systematic review aims to elucidate the bidirectional relationship between puberty and ARD-related outcomes.

**Methods:**

Studies published in English until October 2019 were identified using a systematic search of endocrinology and rheumatology literature. Information was extracted on study design, sample size, demographics, puberty outcome measures, disease outcome measures, and main findings. The methodological quality of the studies included was analysed using the Newcastle-Ottawa Scale (NOS).

**Results:**

Sixteen non-randomised studies reporting on the impact of puberty on ARD outcomes (*n* = 7), ARD impact on puberty-related outcomes (*n* = 8), or both (*n = 1*) have been identified. The impact of puberty on ARD outcomes were investigated in patients with juvenile idiopathic arthritis (JIA)-associated uveitis (*n* = 1), juvenile systemic lupus erythematosus (JSLE) (*n* = 5) or in healthy controls who developed adult-onset SLE (*n* = 1) or had non-specific symptoms (n = 1). The impact of ARD on puberty outcomes was explored in JIA (*n* = 4) and JSLE (*n* = 3). Quality assessment of studies showed a small to moderate risk of bias overall (NOS 4–9/9). Due to large heterogeneity of the studies it was not possible to perform a meta-analysis. Multiple studies reported on delayed puberty in patients with JIA/JSLE, menstrual and hormonal abnormalities, and lower height and weight than controls. Earlier (pre-pubertal) onset of JSLE was correlated with more severe disease and more need for systemic treatment.

**Conclusion:**

A bidirectional relationship exists between puberty and ARDs; however, more and better research is required to elucidate the complexity of this relationship. We propose puberty-related clinical assessments in patients with ARDs, which can improve patient outcomes and facilitate future research.

## Background

Adolescence and puberty are associated with significant physical, physiological, psychological and immunological changes. The timing of puberty is important, as both early and late puberty correlate with adverse health outcomes later in life, such as diabetes and cardiovascular disease [1]. Puberty is initiated and mediated by sex hormones, which also influence the development and regulation of the immune system [2]. Epidemiological studies have shown that pubescent and post-pubescent males and females are at risk of developing autoimmune rheumatic diseases (ARDs) in a sex-biased manner, and that the age at disease onset often differs between sexes [3, 4].

The sex bias in the incidence of autoimmune diseases has long been recognised, with females being at significantly higher risk of developing conditions such as systemic lupus erythematosus (SLE), scleroderma, Sjögren’s syndrome, rheumatoid arthritis (RA), autoimmune thyroid disease, multiple sclerosis (MS), and polyautoimmunity [2, 5]. Furthermore, in many ARDs, the average age of disease onset is significantly earlier in females than in males. The female population undergoes at least two major endocrinological changes in their lifetimes: puberty and the menopause, between which there are constant cyclical hormonal changes, and possibly pregnancy and breastfeeding. Males experience major physiological and psychological changes during puberty as well, but show a gradual decline in hormonal levels after age 40, rather than the abrupt change seen in females during menopause [6]. The endocrinological changes at each of these milestones affect both the innate and adaptive immune systems. T-cell autoimmunity in particular is upregulated post-puberty in animal models [5]; however, there is a limited understanding of the physiological (molecular and cellular) mechanisms for sex-specific immune modulation.

Previous research has observed a relationship between puberty and the onset of various autoimmune conditions. The incidence of SLE, autoimmune thyroid disease, and MS increases in peri- and post-pubescent females, suggesting that sex hormone changes at puberty play an immunomodulatory role in triggering ARD onset and development [5]. The prevalence of juvenile SLE (JSLE) in females has been observed to rise from 6.7 per 100,000 at age seven to 34.6 per 100,000 at age fifteen (versus almost zero per 100,000 at age seven and 7.8 per 100,000 at age fifteen in males) [7]. The female: male incidence ratio in SLE is most pronounced after puberty and before the menopause (from 2:1 to 6:1 pre-puberty, 7:1 to 15:1 post-puberty, and 3:1 to 8:1 post-menopause) [8, 9] suggesting that the increased sex hormone levels present from puberty to the menopause increase susceptibility to an autoimmune state. Similarly, MS is rarely seen pre-puberty, and the female: male ratio of MS onset in pre-pubertal children is near equal. Post-puberty, MS incidence increases in both sexes with a 2.2:1 female: male ratios [10]. Furthermore, there is emerging evidence linking the age at menarche with the risk of autoimmunity in females. Studies have found that an earlier age at menarche increases the risk of RA [11]; later age at menarche decreases the risk of MS [12]; JSLE is associated with a trend for later onset menarche [13]; and the incidence of psoriasis in females increases during the peri-menarchal period [14].

In addition to triggering autoimmunity, sex hormones can influence the outcome of autoimmune diseases. Testosterone is thought to exert a protective effect against the development of autoimmunity. This has been replicated in animal models, which showed protective effects of testosterone in models of both SLE [15] and arthritis [16, 17]. Oestriol, a sex hormone that is only detectable during pregnancy, is thought to have a protective effect in MS, and may account for the reduced relapse rates observed during pregnancy [4]. Previous research has been directed at understanding the impact of hormonal treatment in autoimmune disease: benefits associated with testosterone treatment have been seen in SLE [18] and arthritis [19]; ongoing clinical trials are finding reduced relapse rates with oestriol treatment in MS [20].

Apart from influencing autoimmune disease onset and outcomes, sex hormones impact the physiological development of bones and muscles during puberty [21]. This is frequently accompanied by non-specific musculoskeletal symptoms [22], and changes in bone and muscle biomarker levels [23, 24], further complicating the assessment of ARD-related symptoms in adolescents.

No previous systematic reviews have addressed the impact of puberty on disease outcome measures in autoimmune rheumatic diseases, or the impact of ARDs on puberty-related outcomes. Few studies are available that have looked at the epidemiology of ARDs during adolescence or focused on the gender bias in autoimmunity in adolescent populations. Understanding the interplay between the neuroendocrine and immune systems will provide insights into the pathogenesis of the peri-pubertal onset of ARDs, and may change the clinical approach to treatment of these patients in the long term.

## Aims

With this systematic review we aim to elucidate how rheumatological conditions impact puberty, and how physiological changes of puberty influence incidence and manifestations of autoimmune rheumatic diseases.

The objectives of this systematic review are to:

1. Identify and describe the evidence exploring the bidirectional relationship between puberty and ARDs in adolescence and adulthood;
2. Determine the strength of such evidence.

We hope that the findings of this review will help to inform policy, practice, and future research priorities in the field of puberty and autoimmunity.

## Methodology

### Variables of interest

Our primary outcomes are puberty-related measures in patients with ARDs, such as age at menarche, puberty stages and sex hormone levels. In boys, Tanner stages 4 and 5 are equivalent to the menarche stage in girls. Our secondary variables of interest are ARD-specific outcome measures in peri-pubertal patients, for example disease specific activity and damage scores. We also assessed the strength of evidence found relating to the bidirectional relationship between puberty and ARDs in adolescence and adulthood.

### Search strategy

We used a comprehensive search strategy that aims to be both sensitive and specific. We employed detailed search strategies as deemed appropriate for each database to search Medline, EMBASE and Scopus until October 2019.

An initial search strategy was devised using the MEDLINE thesaurus and indexing system to identify appropriate MeSH headings and key/text words associated with the terms ‘puberty’ AND ‘autoimmune rheumatic diseases’; “puberty” OR “growth spurt” OR “growth retardation” OR “growth delay” AND “juvenile idiopathic arthritis” OR “systemic lupus erythematosus” OR “SLE” OR “dermatomyositis” OR “enthesitis related arthritis” OR “scleroderma” OR “uveitis”; OR “adolescence” AND “juvenile idiopathic arthritis” OR “systemic lupus erythematosus” OR “SLE” OR “dermatomyositis” OR “enthesitis related arthritis” OR “scleroderma” OR “uveitis”. This search strategy has been adapted for use across all included databases as necessary.

The references of papers and review articles were manually checked to ensure inclusion of studies not retrieved through the computerised search method.

### Inclusion criteria

The following studies were included:

1. Studies that include autoimmune rheumatic disease patients of pubertal age, including studies that compare pre- and post-pubertal populations.

2. Prospective and retrospective studies of ARD patients that assess the impact of puberty factors on their disease, or the impact of their disease and/or treatment on puberty outcomes.

The study types included are: observational studies (cohort studies, case-control studies, and cross-sectional studies); experimental studies (randomized controlled trials, controlled clinical trials); case-reports, case-series and abstracts at conferences including subjects with ARDs; studies reporting on incidence/prevalence, clinical and serological ARD features, puberty markers (including clinical assessment, hormone levels or body height/growing patterns), treatment, quality of life, etc.

### Exclusion criteria

The following studies were excluded: studies in other languages than English, review articles, animal models studies, commentaries, editorials, questionnaire studies, duplicates, and papers not relevant to the topic.

### Stages in the literature search

The protocol was finalised in December 2019. The various stages of this literature search were summarised using the Preferred Reporting of Systematic Reviews and Meta-Analysis (PRISMA) flow chart format to visualise the processes and findings of the review.

### Classification of result resources

Data extraction sheets and tables were developed, tailored to the resources found.

### Study selection

Titles and abstracts were screened and independently assessed for eligibility by two reviewers (MK and JG) and the conflicts were resolved by CC. Full-text papers were evaluated in duplicate by AR and HP. Any disagreement regarding their eligibility was resolved by discussion with a third reviewer (CC)**.** The agreement between the reviewers was assessed by Kappa statistic.

### Data extraction and synthesis

Data extraction was independently performed by two reviewers (AR and HP) and discrepancies were resolved through discussion with a third reviewer (CC). We extracted the following information from all the eligible studies: 1) year of publication; 2) country of publication; 3) study design; 4) sample size; 5) demographics of patient group and controls; 6) puberty outcome measures; 7) main findings. In cases where data were not available in the manuscripts, we contacted the authors of relevant papers for additional information.

### Quality assessment

The methodological quality of the studies included was analysed using the validated Newcastle-Ottawa Scale (NOS) for non-randomised studies [25], which assesses the quality of three broad study aspects: the selection of the study groups, their comparability, and the ascertainment of either the exposure or outcome of interest, for case-control or cohort studies respectively. Each of these three items was assessed and graded (1 or 2 points). In this analysis, studies with NOS scores of 1–3, 4–6, and 7–9, were defined as of low, intermediate, and high quality, respectively.

## Results

**Fig. 1:**
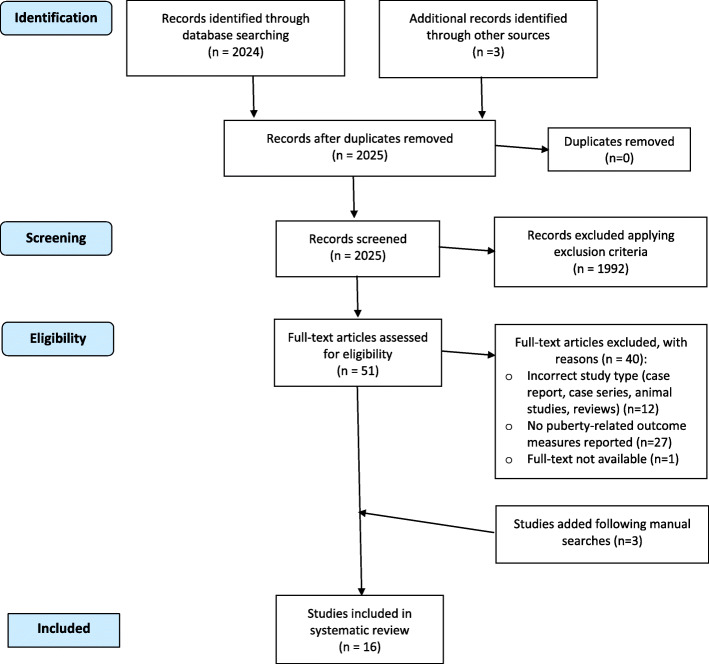
Flowchart of study selection papers

We identified a total of 2027 studies using the search strategy detailed above. After checking for duplicates (*n* = 0), 1992 papers were excluded as they did not fulfil the selection criteria. The remaining 51 full-length articles were screened in detail and a final number of 16 papers were deemed appropriate to be included in the qualitative analysis (Fig. 1). One out of 16 eligible papers reported both on the puberty impact on autoimmune rheumatic disease outcomes and ARD impact on puberty-related outcome measures. The papers were grouped in two tables to enable the separate exploration of the bidirectional relationship between puberty and ARDs.

After scrutinising the types of outcome measures reported in the eligible papers, we concluded that a quantitative analysis (meta-analysis) was impossible because of the high heterogeneity of the reported outcomes and subjects included in the various studies.

The papers reporting the impact of ARDs on puberty-related outcomes (*n* = 9) mainly focused on patients with JIA or juvenile rheumatoid arthritis (JRA) (*n = 5*) and (J) SLE patients (*n = 4*) (Table 1). Of the 8 papers which reported puberty impact on ARD outcomes, 6 papers investigated (J) SLE patients and one paper investigated patients with juvenile idiopathic arthritis (JIA)-associated uveitis (Table 2). We found no eligible studies in patients with dermatomyositis, enthesitis-related arthritis scleroderma, chronic recurrent multifocal osteomyelitis (CRMO) or childhood granulomatosis with polyangiitis. One paper investigated the impact of puberty on the prevalence of ANA positivity and musculoskeletal symptoms, although there was no evidence that patients with these characteristics proceeded to develop an ARD [35]. The papers were published between 1998 and 2019, and most studies were single-centre cohort studies, with limited data on cohort ethnicity; details about ethnicity were only provided in 10 out of 16 papers.

**Table 1:** Impact of autoimmune rheumatic diseases on puberty-related outcomes (listed as per year of publication)

First author, year of publication	Type of study	Country of origin	Participants, disease N (F:M) Age	Controls N (F:M)	Ethnic group	Puberty-related outcomes measured	Results	Quality of the study using the Newcastle-Ottawa scale /9
El Badri et al., 2014 [26]	Cross-sectional	Morocco Single centre	40 (18:22) JIA patients 11 – systemic 9 – oligoarthritis 17 – RF positive polyarthritis 1 – RF negative polyarthritis 1 – enthesitis-related arthritis 1 – psoriatric arthritis Age 11 ± 4.23 years	74 children	Moroccan	Age at puberty onset and menarche	Delayed puberty was observed in JIA patients (15%) vs. controls (1%) (*P* = 0.005). Mean age at menarche was 0.7 years later in patients (12.33) compared to healthy controls (11.62). There was an association between the dose of corticosteroids (*P* < 0.05) and earlier age at the administration of corticosteroids (*P* < 0.05) with delayed puberty in boys.	7/9
Maher et al., 2013 [27]	Cross-sectional	Egypt Single centre	80 (45:35) JRA patients 30 – pauci-articular 33 – polyarticular 17 – systemic Age 13.29 ± 2.42 (range 8–16) years	80 age- and sex-matched healthy controls	Not stated	Age at onset and completion of puberty as measured by Tanner staging G2–5 of genital development, presence/absence of pubic hair, and age of menarche. BMI and weight	Onset of puberty (attainment of stage G2 of genital development) delayed in all JRA groups compared to HC. Age of menarche delayed in all JRA groups compared to HC. Stage G4 not obtained by 16 years in poly-JRA male or female. No JRA groups reached stage G5 by age 16 years. Weight and BMI significantly lower in JRA patients compared to HCs.	7/9
Rygg et al., 2012 [13]	Prospective cohort	Europe Multi centre: PRINTO study (Paediatric rheumatology international trials)	331 (276:55) JSLE patients Median age 13.9 (1st quantile 11.9, 3rd 15.8) years	No; compared to literature or target based on parents	Not stated	Age at puberty onset, menarche and Tanner staging (*n* = 147, only females) Height (*n* = 331) Measurements at baseline, 6, 14 and 26 months	Delayed pubertal onset was found in 15.3% of females and 24% of males. Delayed/absent menarche was found in 21.9% of females. Some degree of delayed pubertal development was found in 36.1% of the females and 44% of the males. Growth failure (defined as parent-adjusted height z-score < − 1.5) ﻿was seen in 16.9% of females and 22.4% of males. Females with prepubertal onset (age < 8 years) had significantly lower parent-adjusted height scores (median baseline z = − 1.99) throughout follow-up. Females with post-pubertal onset (age ﻿ ≥ 13 years) had parent-adjusted height scores near the age-matched reference with no further decrease throughout follow-up. Females with peri-pubertal onset showed a decrease in parent-adjusted height scores over time.	5/9
Aggarwal et al., 2011 [28]	Prospective cohort	North-West India Single centre	70 (0:70) JRA patients 24 – pauci-articular 32 – polyarticular 24 – systemic Age range 9–17 years	134 ‘normal’ boys from a previous study in the same area	Not stated	Age at completion of puberty as measured by Tanner staging G2–5 of genital development, and presence or absence of facial, pubic and axillary hair at 6-month intervals	None of the JRA patients obtained stage G5 of genital development by age 17, compared to the average age of HCs of 15.2 years. Those with systemic JRA didn’t reach stage G4 of genital development by age 17. Puberty initiation (stage G2) was earliest among patients with systemic JRA (10.8 ± 1.3 years), and first appearance of pubic/facial/axillary hair was also earlier among these patients compared to pauci- and poly-JRA.	7/9
Aggarwal et al., 2011 [29]	Prospective cohort	North-West India Single centre	70 (0:70) JRA patients 24 – pauci-articular 32 – polyarticular 24 – systemic Mixed socio-economic background Age range 9–17 years	No; compared to literature data from well-off Indian boys and American boys	Not stated	Growth velocity, measured by body weight and height at 6-month intervals	﻿Patients from all subtypes of JRA measured lighter and shorter than in literature reported well-off Chandigarh, affluent Indian and American boys. Boys with polyarticular and systemic onset JRA were shorter than those with pauci-articular JRA, until 15 years and 12 years, respectively. Onset of peak height velocity in boys with polyarticular JRA was delayed (i.e. 12.5 years) compared to boys with pauci-articular JRA (i.e. 11.5 years). Attainment of Peak Weight Velocity in boys with polyarticular JRA (i.e. 13.5 years) was also delayed by 1 year when compared to those with pauci-articular type (i.e. 12.5 years).	7/9
Medeiros et al., 2009 [30]	Cross-sectional	Brazil Single centre	30 (30:0) JSLE patients Mean age 17.4 ± 3.2 years	30 age- and sex-matched healthy controls	Not stated	Age at menarche, menstrual and hormonal alterations	Age at menarche was higher in JSLE than controls (13. ± 1.4 vs. 11.56 ± 1.5 years, *P* = 0.0008). Menstrual abnormalities and longer length cycles were more frequent in JSLE than controls (63% vs. 10%, *P* = 0.0001; 23% vs. 0%, *P* = 0.0105, respectively). The median of FSH was significantly higher in patients with JSLE compared with controls (4.6 vs. 3.4 IU/L, *P* = 0.0207), and the median of progesterone was lower (32.5 vs. 70 ng/mL, *P* = 0.0033). The median of LH was lower in patients with JSLE with menstrual abnormalities versus normal cycles (2.9 vs. 5.5 IU/L, *P* = 0.019).	7/9
Silva et al., 2002 [31]	Retrospective cohort	Brazil Single centre	23 (23:0) SLE patients Age range 16.75–22.83 years	No; compared to historical healthy control data on 2578 Brazilian adolescents	Not stated	Gonadal function and age of menarche	Mean age of menarche (13.5 ± 1.4 years) in SLE patients was greater than that of healthy Brazilian adolescents (12.5 ± 1.3 years; *P* = 0.0002). ﻿70% of SLE patients showed normal gonadal function.	6/9
Gutiérrez-Suárez et al., 2006 [32]	Cross-sectional	39 Countries across Europe, North & South America, Asia and Oceania Multi-Centre	1015 (﻿846:169) JSLE patients Mean age ﻿15.9 ± 4.1 (range 2.4–34.8) years	No; compared to mean for age according to literature	Not stated	Growth (*n* = 943) defined by height Puberty stage (*n* = 556) defined by development of secondary sexual characteristics	15.3% had growth failure (height > 2 SD below mean for age) 11.3% had delayed puberty (secondary sexual characteristics > 2 SD below the mean for age by Tanner staging). ﻿The frequency of both growth failure (*P* < 0.001) and delayed puberty (P = 0.02) increased significantly with the increase in disease duration.	4/9
Fraser et al., 1988 [33]	Retrospective cross-sectional	Massachusetts USA Two centres	68 (68:0) JRA patients 35 – pauci-articular 18 – polyarticular 15 – systemic Age not reported	46 patient sisters (all without JRA) (one a mono-zygotic twin)	Caucasian	Association between age at disease onset, JRA diagnosis on age of menarche	Girls with JRA have later mean age of menarche 13.2 years vs. 12.5 years for siblings (*p* = 0.015) irrespective of recorded steroid use (in 29.4% JRA patients) Age of disease onset was not an important predictor of age of menarche.	4/9

**Table 2:** **Impact of puberty on autoimmune rheumatic disease-related outcomes (listed as per year of publication)**

First author, year of publication	Type of study	Country of origin	Participants, disease N (F:M) Age	Study groups, comparison	Ethnic group	Disease outcomes measured	Results	Quality of the study using the Newcastle-Ottawa scale /9
Abdawani et al., 2019 [34]	Retrospective cohort	Oman Single Centre	103 JSLE patients 39 (27:12) pre-pubertal onset Mean age 5.12 ± 1.98 years 29 (24:5) pubertal onset Mean age 10.8 ± 0.99 years 35 (32:3) post-pubertal onset Mean age 15.3 ± 1.59 years	JSLE patients stratified based on pubertal status at disease onset	Arab	Association between clinical manifestations and antibody frequencies in JSLE patients stratified based on pubertal status at disease onset	Increased renal disease in pre-pubertal compared to pubertal and post-pubertal groups, respectively (51% vs 23% vs 20%; *P* = 0.039) Pre-pubertal onset JSLE had a higher incidence of cutaneous manifestations than the post-pubertal group (74% vs 46%; *P* = 0.029) Pre-pubertal onset JSLE had increased frequencies of anti-cardiolipin antibodies (47%), anti-glycoprotein antibodies (42%), ANCA (62%), and low complement levels (97%) Pre-pubertal group also has the lowest frequency of positive SSA antibodies (18%) and SSB antibodies (5.1%)	7/9
Sperotto et al., 2014 [35]	Prospective cohort	Italy 4 centres	261 (137:124) baseline healthy children 184 pre-pubertal 77 pubertal Mean age 10.6 (range 8–13) years	3 year-follow-up of pre-pubertal and pubertal children with ANA positivity (*n* = 32) or chronic musculoskeletal pain (*n* = 77) at baseline	Not stated	Musculoskeletal chronic pain assessed by rheumatologic examination, ANA, dsDNA and ENA antibody titres	Already positive ANA titres increased in value during puberty (*P* = 0.002). ANA positivity has no relationship with chronic non-inflammatory musculoskeletal pain. After puberty, more females than males were ANA positive (50% vs 28%)	5/9
Hoeve et al., 2012 [36]	Retrospective cohort	The Netherlands Multi centre	62 (40:22) patients with JIA-associated uveitis Mean age at diagnosis of uveitis 4.9 ± 1.7 years	Follow-up of patients before, after and during puberty	Not stated	Influence of puberty on the long-term course of uveitis	Similar incidence of cystoid macular oedema and papillitis between pre-puberty and in-puberty, but more boys developed ocular hypotony during puberty compared to pre-puberty (*P* = 0.026) More systemic treatment for uveitis was required for girls during puberty compared to pre-puberty (*P* < 0.001) ﻿JIA-associated uveitis encompasses a biphasic course: a high initial disease activity, followed by a quiet stage and a new wave of activity during early teenage years.	6/9
Hui-Yuen et al., 2011 [37]	Retrospective cohort	USA Single centre	34 (27:7) pre-pubertal JSLE 34 (27:7) pubertal JSLE Age at diagnosis Pre-pubertal 8.7 ± 2.6 Post-pubertal 14.8 ± 2.0 years	Pre pubertal (Tanner I-II) patients were matched to pubertal (Tanner III-V) patients	African-American, Asian, Caucasian, Hispanic, Other	Clinical and lab characteristics, medication use, organ involvement, paediatric intensive care unit (PICU) admissions, disease activity.	Early onset JSLE (pre-pubertal): -required greater number of PICU admissions (18 vs. 5, *P* = 0.01) -required higher daily steroid dose (0.6 mg/kg prednisone-equivalent versus 0.2 mg/kg, *P* < 0.05) -received cyclophosphamide earlier in their disease course (mean 13.7 versus 19.9 months, *P* < 0.001)	6/9
Descloux et al., 2009 [38]	Retrospective cohort	France Single centre	56 (39:17) JSLE patients Mean age at disease onset 12.6 ± 3.2 years (median 13 years)	JSLE patients with damage were compared with those without damage	Caucasian, Afro-Caribbean, Asian, Middle Eastern	Damage as measured by SDI or death.	The risk of damage (SDI ≥ 1) significantly decreased when age at disease onset increased (89% in pre-pubertal JSLE, 57% in peri-pubertal JSLE and 38% in post-pubertal JSLE) (*P* = 0.032)	7/9
Costenbader et al., 2007 [39]	Prospective cohort	UK NHS (Nurses’ Health Study) and NHSII UK national data base of female nurses	238,308 (238,308:0) women from two national cohorts 121,700 ages 30–55 years 116,608 ages 25–42 years	Nurses who developed SLE were compared to the ones who did not	Caucasian (> 97%), African, and Hispanic.	Development of SLE.	262 incident cases of SLE were confirmed. In multivariable models adjusted for reproductive and other risk factors, age less than 10 years at menarche (pooled RR 2.1, 95% confidence interval [95% CI] 1.4–3.2) was associated with development of SLE.	9/9
Pluchinotta et al., 2007 [40]	Retrospective cohort	Italy Single centre	42 JSLE patients + 11 infantile JSLE cases reported in literature 13 (1.2:1) infantile (age of diagnosis < 2 years old) 11 (1.2:1) pre-pubertal (age of diagnosis 2–10 years) 29 (6.3:1) post-pubertal (age of diagnosis 10–16 years)	Comparison between infancy, prepubertal and post-pubertal JSLE patients	Caucasian (92%), Indian, African American	Prevalence and severity of organ involvement, blood count and auto-antibodies.	Infantile JSLE was more severe than childhood SLE with a higher prevalence of cardiovascular (*P* < 0.05) and pulmonary involvement (*P* < 0.05), anaemia (*P* < 0.05) and thrombocytopenia (*P* < 0.01) Post-pubertal patients had a higher frequency of musculoskeletal involvement (*P* < 0.005) and leukopenia.	6/9
Silva et al., 2002 [31]	Retrospective cohort	Brazil Single centre	23 (23:0) SLE patients Age range 16.75–22.83 years	No; compared to historical healthy control data on 2578 Brazilian adolescents	Not stated	Disease duration, cumulative prednisone dose, disease activity measured by SLEDAI	Delay in menarche correlated positively with disease duration (*P* = 0.0085) and cumulative dose of prednisolone prior to menarche (*P* = 0.0013). Gonadal function did not correlate with mean SLEDAI score.	6/9

The Cohen’s Kappa Statistic value calculated for the agreement reached by the manual screening of the paper abstracts was 0.69 (95%CI 0.682–0.71), while the screening of the full-text papers reached an agreement of 0.81 (95%CI 0.79–0.82).

The quality assessment of the included studies according to the Newcastle-Ottawa Scale, assessing criteria such as selection, comparability and outcome, ranged from 4 to 9/9, showing a small to moderate risk of bias overall (Tables 1 and 2).

### Impact of autoimmune rheumatic diseases on puberty-related outcomes

The disease outcome measures investigated in JIA/JRA studies included cumulative dose of steroids, disease duration, disease activity, height, weight and age [26], as well as diagnosis [26–28, 33], type of JIA (systemic) [28] and age at disease onset [33]. Studies investigating the impact of JSLE on puberty were exclusively focused on evaluating the impact of being diagnosed with JSLE on puberty-related outcomes [13, 30, 31]. The following aspects of puberty were investigated: age at puberty onset; Tanner staging G2–5 of genital development [28]; presence or absence of facial, pubic and axillary hair [28]; age at menarche [30, 31, 33]; menstrual abnormalities and length of menstrual cycles, follicular stimulating hormone (FSH) and luteinizing hormone (LH) levels [30].

All the studies investigating the impact of JIA/JSLE on puberty reported some extent of delayed puberty in children with these conditions compared to healthy controls. Aggarwal et al. reported earlier puberty onset (Tanner stage G2 and onset of pubic/facial/axillary hair) in boys with systemic JIA compared to oligo- and polyarticular JIA [28], although despite this, the completion of puberty (defined as Tanner stage G5 achievement) was delayed in all male JIA patients compared to healthy controls. One study investigated the impact of JRA diagnosis on BMI and height, and found both to be significantly lower in JRA patients compared to age-matched healthy controls [27]. Although there is evidence of growth delay associated with JIA in both boys and girls, the JRA clinical phenotype had a differential impact on growth in peri-pubertal Indian boys: boys with polyarticular and systemic onset JRA were shorter than those with pauci-articular JRA, until 15 years and 12 years of age, respectively [29]. Similarly, adolescent JSLE patients experienced delayed puberty and growth retardation when compared to healthy controls as reported in the literature, and both correlated with the disease duration [32]. The impact of ARD-related treatment on puberty was sparsely evaluated: one study in JIA showed a significant association between both the dose and an earlier age at administration of corticosteroids with delayed puberty in boys [26], while an older study did not show any impact of steroid use in girls with JRA on age at menarche compared to healthy controls [33]. One study investigated additional puberty-related outcomes, and found that menstrual and hormonal abnormalities were significantly more common in patients with JSLE than in healthy controls [30].

### Impact of puberty on autoimmune rheumatic disease-related outcomes

The studies investigating the impact of puberty on autoimmune rheumatic disease manifestations and severity included the following puberty parameters: Tanner stages [34, 36–38, 40], age at menarche [31, 39], female reproductive factors [39] and gonadal function [31]. One study focused on patients with JIA-associated uveitis [36], all the remaining studies investigated patients with JSLE or healthy controls who developed adult-onset SLE.

The impact of age at onset and puberty on JSLE severity was consistent across studies, showing similar trends of more severe disease in patients with earlier onset (pre-pubertal) compared to post-pubertal patients, irrespective of outcome measures used (renal, cardiovascular, pulmonary and haematological involvement; paediatric intensive care unit [PICU] admissions; steroid and cyclophosphamide use; autoantibodies and complement C3 levels, and accrued lupus-related damage). Of note, none of the JSLE studies investigating the impact of puberty evaluated overlapping outcome measures to allow for cross-validation of findings. One study showed, through multivariate analysis, that an age of less than 10 years at menarche was associated with the risk of SLE development later in life [39]. Another study [31] found that delayed onset of menarche related strongly with both JSLE disease duration and cumulative prednisolone dose pre-menarche. This may be because both chronic disease as well as chronic use of corticosteroids can suppress growth, which can delay menarche and slow down puberty progression.

We identified only one study investigating puberty-related outcomes in ARD patients stratified based on sex [36]. This study found increased incidence of ocular hypotony during puberty in boys with JIA-associated uveitis, and also found that pubertal females more frequently required systemic treatment for uveitis compared to either sex pre-puberty. The same study observed a biphasic course in JIA-associated uveitis in both boys and girls: a high activity at diagnosis, followed by a reduction, and a new wave of activity during early teenage years [36]. However, the second activity wave cannot be linked to pubertal changes directly, as the authors do not provide puberty measurements or stages.

## Discussion

Adolescence is associated with significant changes that are initiated and mediated by sex hormones. Recent efforts collecting detailed prospective data help to understand the impact of hormonal changes during the transition from childhood to adolescence on psychological and physical health [41]. During and after puberty, ARDs are more common in females than in males. Research suggests a potential role for sex hormones in triggering autoimmune processes, and in influencing the outcomes of ARDs.

With this systematic review we set out to describe the evidence supporting the bidirectional relationship between puberty and ARDs. It has highlighted the large heterogeneity of the available literature. Overall, the studies identified had moderate quality, and investigated, with few exceptions, cohorts of a relatively small sample size. The majority were retrospective studies and provided insufficient detail on the management of missing data. All studies focussed on JIA/JRA or (J)SLE. Because of the heterogeneity of disease-specific outcome measures reported, there was no scope to perform a meta-analysis. Despite the puberty-related outcomes being less heterogeneous – the majority of the studies reported the age at menarche or completion of various Tanner stages – a meta-analysis of the impact of one specific ARD on puberty could not be performed, due to the variation of result reporting and the small number of studies per disease. Despite being aware that puberty has significant impact on growth, and that the growth spurt is a useful clinical puberty surrogate outcome for boys [42], we identified only one paper assessing the growth velocity delay during puberty in boys with JIA [29]. However, there are many studies in JIA showing reduced attained height in JIA patients over age 18 [43], as well as reduced height 3 years after diagnosis (median age 10.4) in children with JIA compared to healthy controls [44]. Similarly, there is evidence of growth retardation at 2-year follow-up in patients with JSLE, which prompted the proposal of including growth failure and delayed puberty in the paediatric version of the Systemic Lupus International Collaborating Clinics/American College of Rheumatology Damage Index (SDI) [32]. It is unknown, however, whether growth retardation in young people with ARDs is a symptom of delayed puberty, or an effect of chronic illness in combination with growth-inhibiting medications, such as corticosteroids [43].

Although a quantitative analysis was not possible, there were clinically relevant results worth mentioning. For example, a delay in puberty in patients with JIA/JSLE compared to controls was reported in all studies investigating the impact of these diseases on puberty outcomes. Lower height and weight than healthy controls, and menstrual and hormonal abnormalities, were also seen in ARD patients. Some evidence suggests the delay in puberty is positively correlated with (cumulative) corticosteroid dose, though further research is needed to confirm this; it is likely that the correlation reflects an indirect effect of disease severity, and delayed growth caused by corticosteroids [43, 45]. Clarifying this link is especially relevant considering that multiple articles reported more severe disease, and more need for (systemic) treatment, in children with pre-pubertal diagnosis of ARD, as compared to post-pubertal diagnosis.

**Fig. 2:**
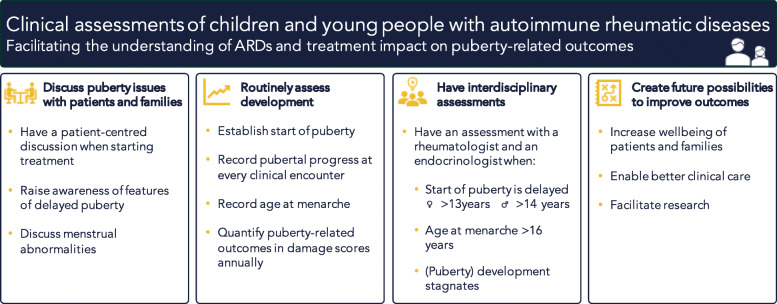
Suggested clinical assessments of children and adolescents with autoimmune rheumatic diseases

Although our literature search did not identify papers assessing other ARDs with onset around the time of puberty, such as CRMO (age at onset 7–12) [46] or childhood granulomatosis with polyangiitis (median age at onset 11.7 years) [47], we recognise that the assessment of puberty-related outcomes is relevant for a larger group of rheumatic conditions than the ones captured in this review. In light of the limited quality of evidence in this systematic review, we want to propose a set of feasible clinical assessments of children and young people with autoimmune rheumatic diseases. These recommendations, based on the preceding results and the experience of our multidisciplinary clinical team, can be recorded at hospital visits to facilitate the understanding of disease and treatment impact on puberty-related outcomes. We suggest the following: 1) to establish start of puberty in all patients to ensure appropriate action can be taken if it is delayed (age > 13 years in girls and > 14 years in boys); 2) to record age at menarche in every patient to establish that it is not delayed (age > 16); 3) to record pubertal progress – by history/self-assessment and growth chart, and by clinical examination in case of concerns – at every clinical encounter until the completion of puberty, in accordance with the Childhood and Puberty Close monitoring Charts [48]; 4) to discuss menstrual abnormalities; 5) to have patient-centred discussions around starting treatments that are likely to (indirectly) impact puberty, such as steroids or cyclophosphamide, and ensure pubertal development is monitored in patients started on such treatments; 6) to propose endocrinology-rheumatology interdisciplinary assessments in selected cases of delayed puberty; 7) to raise patient/family awareness of features of delayed puberty; 8) to quantify puberty-related outcomes in damage scores annually, e.g. growth retardation, delayed puberty, or infertility (see Fig. 2). Quantifying outcomes will greatly improve care: it allows for close monitoring and provides a starting point for conversation – especially when including self-assessment – involving young people in their own care.

These recommendations are both in line with the ﻿EULAR/PReS recommendations for the transitional care of young people with juvenile-onset rheumatic diseases [49], and with the general notion that routine developmental assessment is a core part of adolescent health care [41, 43]. This supports our opinion that evidence-based knowledge is needed to improve outcomes.

Clinicians, patients and families will benefit from increased awareness of the relationship between puberty and ARDs. Not only will our recommended assessments inform individualised flare management during puberty; they will also facilitate future research into treatments that minimise the negative impact of ARD on pubertal development. Shedding light on the complex but important relationship between puberty and autoimmune rheumatic diseases allows young patients to have what all young people want: a chance to develop into the best version of themselves, without limitations.
